# Metformin Resistance Is Associated with Expression of Inflammatory and Invasive Genes in A549 Lung Cancer Cells

**DOI:** 10.3390/genes14051014

**Published:** 2023-04-29

**Authors:** Dong Soo Seo, Sungmin Joo, Seungwoo Baek, Jaehyeon Kang, Taeg Kyu Kwon, Younghoon Jang

**Affiliations:** 1 Department of Biology and Chemistry, Changwon National University, Changwon 51140, Republic of Korea; mizar1105@naver.com (D.S.S.);; 2 Department of Immunology, School of Medicine, Keimyung University, Daegu 42601, Republic of Korea

**Keywords:** metformin, drug resistance, A549 lung cancer cells, proinflammatory genes, invasive genes

## Abstract

Metformin, the most commonly used drug for type 2 diabetes, has recently been shown to have beneficial effects in patients with cancer. Despite growing evidence that metformin can inhibit tumor cell proliferation, invasion, and metastasis, studies on drug resistance and its side effects are lacking. Here, we aimed to establish metformin-resistant A549 human lung cancer cells (A549-R) to determine the side effects of metformin resistance. Toward this, we established A549-R by way of prolonged treatment with metformin and examined the changes in gene expression, cell migration, cell cycle, and mitochondrial fragmentation. Metformin resistance is associated with increased G1-phase cell cycle arrest and impaired mitochondrial fragmentation in A549 cells. We demonstrated that metformin resistance highly increased the expression of proinflammatory and invasive genes, including *BMP5*, *CXCL3*, *VCAM1*, and *POSTN*, using RNA-seq analysis. A549-R exhibited increased cell migration and focal adhesion formation, suggesting that metformin resistance may potentially lead to metastasis during anti-cancer therapy with metformin. Taken together, our findings indicate that metformin resistance may lead to invasion in lung cancer cells.

## 1. Introduction

Metformin is a biguanide compound derived from the galegine found in *Galega officinalis* [1]. It is a guanidine derivative widely used since the 1950s for its anti-hyperglycemic effect. The mechanism of action of metformin in cells has long been unknown; however, it has become the most prescribed antidiabetic drug worldwide because it is inexpensive, safe, and effective [2]. Interestingly, recent studies have found that metformin can regulate AMP levels and the 5′-adenosine monophosphate-activated protein kinase (AMPK) pathway by targeting mitochondrial complex I and lysosomal presenilin enhancer protein 2 (PEN2) [3,4]. These mechanisms may be metformin’s primary mode of action in lowering hepatic glucose output. As a result, metformin can systemically inhibit hepatic glucagon signaling and gluconeogenesis [5]. In addition to its hypoglycemic and antidiabetic effects, many studies have reported that metformin may have various effects on obesity, aging-associated inflammation, aging, and cancer [6,7,8].

Metformin may affect tumorigenesis in patients without diabetes and has antidiabetic effects in patients with diabetes. Diabetes mellitus has been associated with a 1- to 2-fold increase in cancer incidence [9], and many studies have reported that metformin treatment could reduce cancer drug resistance [10]. For example, metformin may reduce the risk of breast, lung, colon, liver, and ovarian cancers and chemotherapy resistance in patients [11,12]. However, current studies have reported no significant or controversial effects on several cancer mortalities [13], indicating that the anti-tumor mechanism of metformin remains elusive. Many studies have focused on the anti-tumor or combined treatment effects of metformin; however, metformin may promote cancer progression by way of an unknown mechanism [14]. This may be a crucial part of the unexpected side effects of metformin resistance in anti-cancer therapy. In this study, we aimed to establish and characterize metformin-resistant A549 human lung cancer cells (hereafter referred to as A549-R cells) to determine the side effects of metformin resistance.

## 2. Materials and Methods

### 2.1. Antibodies and Chemicals

The antibodies were procured from Cell Signaling Technology (Danvers, MA, USA), BioLegend (San Diego, CA, USA), Abcam (Cambridge, UK), Santa Cruz Biotechnology (Dallas, TX, USA), BD Biosciences (San Jose, CA, USA), and GeneTex (Irvine, CA, USA, Table S1). Metformin (D150959) and propidium iodide (PI, 81845), Hoechst 33342 (H3570), rapamycin (553210), SB202190 (S7067), and Gefitinib (SML1657) were purchased from Sigma-Aldrich (Burlington, MA, USA) and Thermo Fisher Scientific (Waltham, MA, USA), respectively. Fluor^TM^ 594 phalloidin (RCS2314) and MitoTracker Green fluorescent dye (RMS1101) for mitochondrial visualization were purchased from BioActs (Inchon, Korea).

### 2.2. Cell Culture and Viability Assay

A549, H460, HeLa, and MDA-MB-231 cells were obtained from American Type Culture Collection (Manassas, VA, USA) and cultured in growth medium (RPMI1640 with 10% fetal bovine serum (FBS) and 1% antibiotic-antimycotic) (Thermo Fisher Scientific). A549-W cells were cultured with metformin to establish A549-R. The concentration of metformin was increased by 0.5 mM every 2 weeks until it reached 8 mM, which is half of the maximal inhibitory concentration (IC50) for metformin in A549-W cells. The cells were seeded in 96-well plates and incubated under the indicated experimental conditions for the cell viability assay. The MTT reagent (CellTiter 96^®^, Promega) was added to each plate and incubated for 4 h at 37 °C according to the manufacturer’s instructions. Absorbance at 470 nm was detected using a MULTISKAN SkyHigh spectrophotometer (Thermo Scientific). The cells were seeded in 6-well plates and counted by trypan blue (Thermo Fisher Scientific) exclusion assay using a LUNA-II™ cell counter (Logos Biosystems) to assess the cell growth rates.

### 2.3. Cell Cycle Analysis

Cells harvested in phosphate-buffered saline (PBS) were fixed in 70% ethanol for 30 min on ice. They were then washed with PBS and incubated with 100 µg/mLRNase A for 20 min. Thereafter, 10 µg/mL propidium iodide was added, and the cells were analyzed using a flow cytometer (BD Bioscience FACSCalibur, Becton Dickinson), following the manufacturer’s instructions. The results were quantified using WinMDI 2.8 software.

### 2.4. Western Blot Analysis

Western blot analysis of whole-cell lysates or histone extracts was performed as previously described [15]. Briefly, the protein lysates were boiled in NuPAGE™ LDS loading buffer (Thermo Fisher Scientific), size-separated on SDS-PAGE, and transferred to PVDF membranes. The protein bands were visualized using chemiluminescence ECL solution (Thermo Fisher) on an iBright™ 1500 Chemidoc machine (Thermo Fisher), then densitometry was analyzed by ImageJ’s gel analysis software.

### 2.5. RNA-Seq and Data Analysis

mRNA-seq and data analyses were performed as previously described [15]. Briefly, mRNA from total RNA was purified using poly T oligo-attached magnetic beads, and mRNA libraries were prepared using the NEBNext Ultra II RNA library preparation kit for Illumina (NEB), according to the manufacturer’s protocol. Qubit and bioanalyzer were used for size selection, quantification, and library quality control. Quantified libraries were sequenced on an Illumina platform (NovaSeq 6000) according to the manufacturer’s instructions. Raw sequencing data were aligned to the human reference genome (GRCh38/hg38) using Hisat2 v2.0.5. For quantification, featureCounts v1.5.0-p3 was used to count the read numbers mapped to each gene, and the fragments per kilobase of exon per million mapped fragments (FPKM) of each gene was calculated based on the length of the gene and read count mapped to this gene (Table S2). Differentially expressed genes (DEGs) in the two conditions/groups were analyzed using the edgeR package (3.22.5). The ClusterProfiler R package was used for enrichment analysis (Gene Ontology and Kyoto Encyclopedia of Genes and Genomes (KEGG) pathway). DEG analysis was performed using an adjusted *p*-value (Padj) <0.05 as a 2-fold cutoff.

### 2.6. Reverse Transcription-Quantitative PCR (RT-qPCR)

Total RNA was extracted using TRIzol (Thermo Fisher Scientific) and reverse-transcribed using a ProtoScript II First Strand cDNA Synthesis Kit (NEB), per the manufacturer’s instructions. Gene expression was determined using the QuantStudio™ 6 (Thermo Fisher) qPCR machine using the Luna^®^ qPCR Master Mix (NEB) according to the manufacturer’s protocol. The SYBR Green primers used are shown in Table S3.

### 2.7. Cell Migration Assay

For wound healing assay as migration ability, the cells were seeded in 6-well plates and incubated for 24 h. The cells were scratched using a p-200 pipette tip and then gently washed with PBS. Cells were incubated with RPMI medium containing 1% FBS for the indicated durations. After 0, 24, and 48 h, cells were imaged, and cell migration was determined by the rate of change in the scratched area using ImageJ software 1.53t.

### 2.8. Immunofluoresce Assay

For measurement of focal adhesion formation, cells were fixed in 10% formalin and permeabilized with 0.25% triton X-100. After blocking with 3% BSA, cells were incubated with primary antibody and fluorescence dye-conjugated secondary antibody, or phalloidin containing hoechst 33342, and mounted with an anti-fading mounting solution (Dako). Cell images were acquired using a fluorescence microscope (CELENA^®^ S Digital Imaging System; Logos Biosystems). Information on antibodies and chemicals is described in Section 2.1.

### 2.9. Statistics

The results are expressed as the mean ± SEM from at least three independent experiments. The statistical significance of differences was determined using analysis of variance or two-tailed Student’s *t*-test in Prism GraphPad software. Statistical significance was set at *p* < 0.05 or 0.01.

## 3. Results

### 3.1. Establishment of A549-R Cells

**Figure 1:**
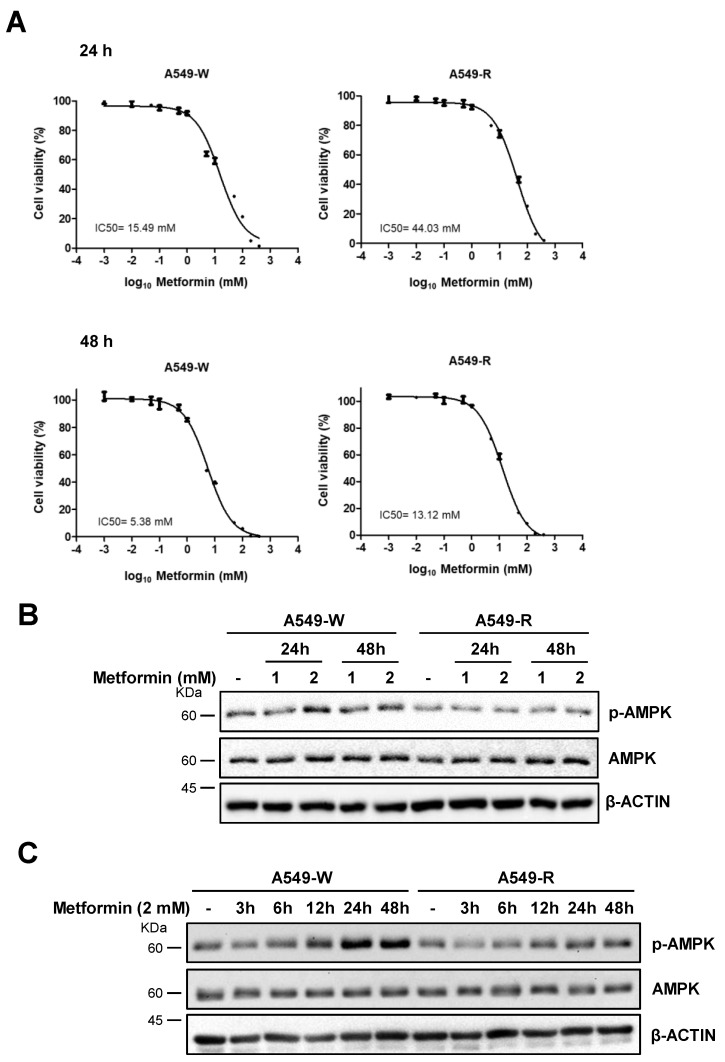
Establishment of metformin-resistant A549 human lung cancer cells (A549-R cells). A549-R was established by cultured A549-W cells with metformin until the half-maximal inhibitory concentration (IC50) was reached. (**A**) A549 cells were cultured with or without increasing concentrations of metformin (until it reached IC50 concentration) for up to 8 months. IC50 calculations were performed using an MTT cell viability assay under metformin treatment for 24 or 48 h. Quantitative data are presented as means ± SEM (*n* = 4). (**B**,**C**) WT (A549-W) and A549-R lines were treated with metformin at the indicated concentrations (**B**) or for the indicated incubation times (**C**), followed by Western blot analysis using the antibodies indicated on the right. β-ACTIN was used as a loading control.

Metformin-resistant cells were established using the A549 human lung cancer cell line to investigate metformin resistance in cancer cells. A549 cells were cultured with metformin following an established protocol [16]. The half-maximal inhibitory concentration (IC50) for A549-R cells was calculated using an MTT cell viability assay. We observed an approximately 2–3-fold increase in IC50 of 24–96 h metformin treatment in A549-R cells compared to control wild-type A549 cells (hereafter referred to as A549-W) (Figure 1A and Figure S1). A549-R cells did not show cross-resistance to another representative chemotherapeutic drug, the tyrosine kinase inhibitor gefitinib (Figure S2) [17]. Other representative cancer cell lines, H460, MDA-MB-231, and HeLa, had relatively high IC50s of 48 h metformin treatment compared to A549 cells (Figure S3), indicating that A549 is a suitable model for metformin drug-resistant cancer cell line. Metformin functions by activating the AMPK kinase [5,18,19], and we performed Western blot analysis for AMPK activation by way of phosphorylation of Thr172 in the activation loop of the catalytic α-subunit by upstream kinases to confirm metformin resistance. A549-R cells exhibited reduced Thr172 phosphorylation levels of AMPK in a concentration- and time-dependent manner (Figure 1B,C). These data suggested that metformin resistance inhibited AMPK activation in A549 lung cancer cells.

### 3.2. Gene Expression Profile of A549-R upon Metformin Treatment

**Figure 2:**
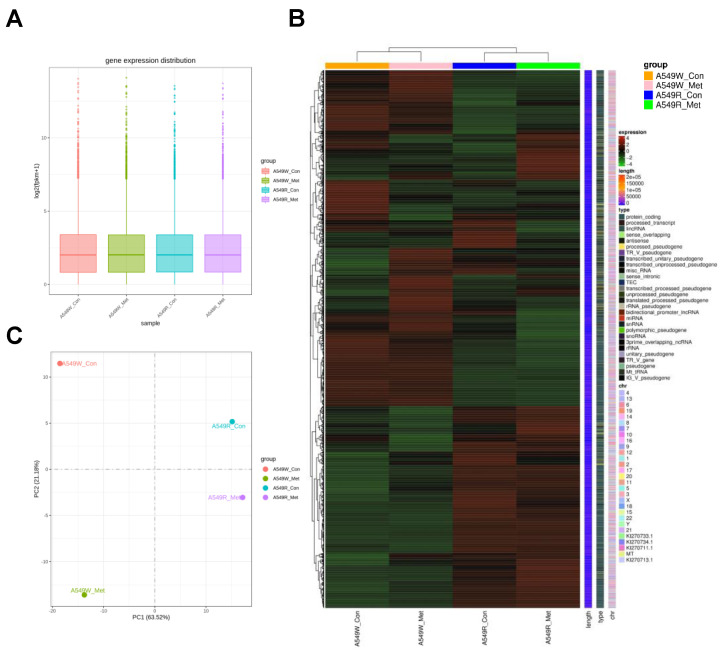
Transcriptome analysis of A549-R cells. RNA-seq analysis was performed to determine the gene expression profiles of A549-R following metformin treatment. (**A**–**C**) For the overall RNA-seq data quality, gene expression distribution (**A**), heatmap (**B**), and principal component analysis (**C**) are shown. Heatmap data is clustered using FPKM values. The gene expression levels, chromosomal locations, lengths, and biotypes of genes are shown to the right and in Table S1 (FPKM value).

**Figure 3:**
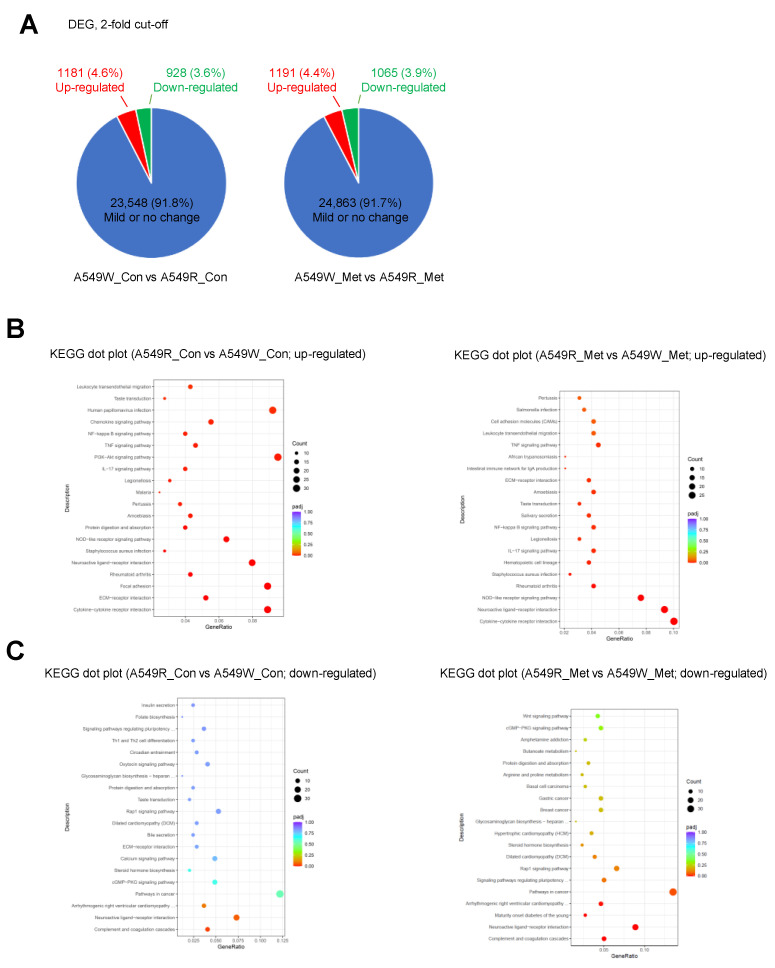
Differentially expressed gene analysis from the transcriptome of A549-R cells. (**A**) Venn diagrams by EdgdR DEG analysis represent the number of all DEGs in A549-W cells compared to A549-R with or without 2 mM metformin treatment for 24 h. The cutoff is two-fold. The *p*-value adjusted (Padj) is <0.05. (**B**,**C**) KEGG enrichment scatter plots of the upregulated (**B**) and downregulated (**C**) gene groups. The gene ontology (GO) dot plot analysis is shown in Figure S3. The size of the dots indicates the number of genes annotated in KEGG pathway or GO analysis. Colors from red to purple indicate the significance of enrichment.

To better assess the effect of metformin resistance on cancer cell gene expression, we performed RNA-seq analysis of A549-R and A549-W upon metformin treatment. Box plots (Figure 2A), inter-sample correlation (Figure S4), and heat maps (Figure 2B) were generated for clustered mRNA transcriptomes from RNA-seq raw data. Principal component analysis (PCA) of A549-R with A549-W showed differential gene expression patterns with and without metformin treatment (Figure 2C). Using a 2-fold cutoff, we defined DEGs that were upregulated (without metformin; 4.6% 1181/25,657) or downregulated (with metformin; 4.4% 1191/27,119) by metformin resistance with or without metformin treatment (Figure 3A). Interestingly, KEGG and GO dot plot analyses revealed that gene groups upregulated by metformin resistance were strongly associated with cytokine-cytokine receptor interaction, focal adhesion, and extracellular matrix (ECM) (Figure 3B and Figure S5). In contrast, we identified a few significant KEGG pathways among the downregulated genes (Figure 3C). Together, these data indicated that metformin resistance in cancer cells is likely related to inflammatory cytokine genes and cellular motility genes involved in invasion and migration.

### 3.3. The Expression of Proinflammatory and Cell Adhesion Genes Is Highly Induced in A549-R

**Figure 4:**
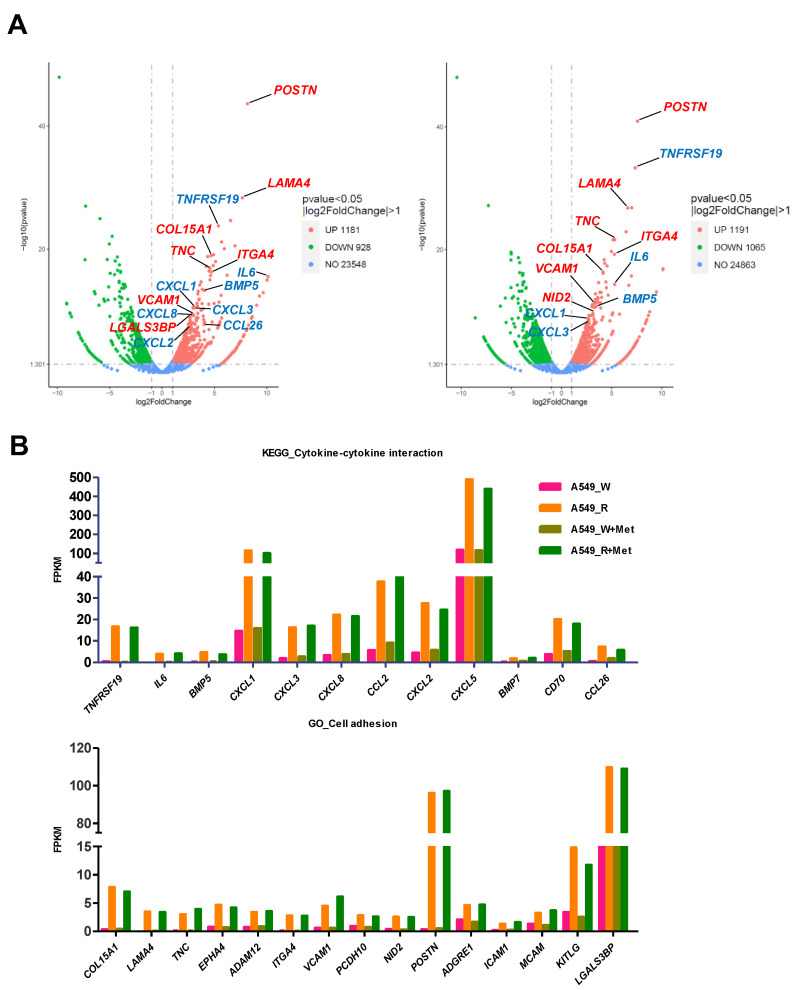
Expression of proinflammatory and cell adhesion genes is highly induced in A549-R cells. (**A**) Volcano plot showing the number of DEGs, including unchanged (blue dot), downregulated (green dot), and upregulated (red dot) genes, for each comparison of the A549-W and A549-R lines with (right panel) or without (left panel) 2 mM metformin treatment for 24 h. (**B**) Representative FPKM values of cytokine-cytokine interaction and cell adhesion gene group in A549-R upon metformin treatment.

**Figure 5:**
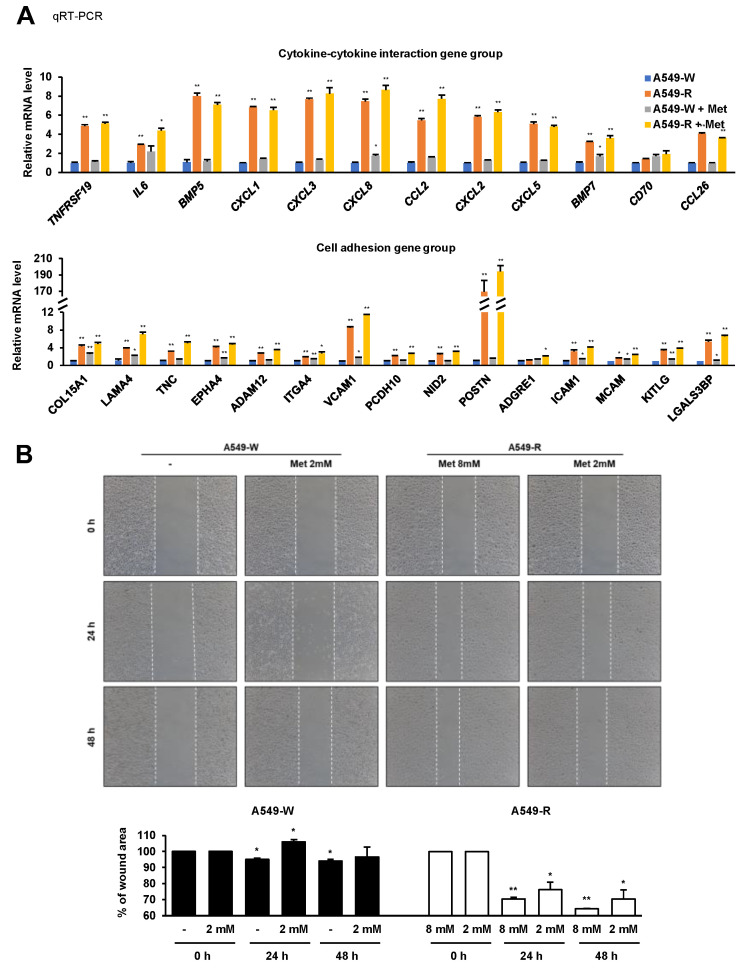
A549-R cells exhibit increased cell migration. (**A**) RT-qPCR of proinflammatory and cell adhesion genes expressed in both A549-W and A549-R cells. Quantitative data are presented as means ± SEM. Statistical comparison between groups was performed using Student’s *t*-test. The A549-W line was compared to the A549-R line in three biological replicates (*n* = 3). * *p* < 0.05, ** *p* < 0.01. (**B**) A wound-healing assay was performed to test the effect of metformin resistance on cell migration. After seeding 4 × 10^5^ cells into 6-well plates, a wound was incised in the center of the plate, followed by washing to remove the detached cells. Phase-contrast images of the wounded area were captured using a microscope, and the relative wound healing rates were calculated at the indicated concentrations or incubation time points. The A549-W and A549-R lines were compared with each control group line in three biological replicates (*n* = 3). * *p* < 0.05, ** *p* < 0.01.

**Figure 6:**
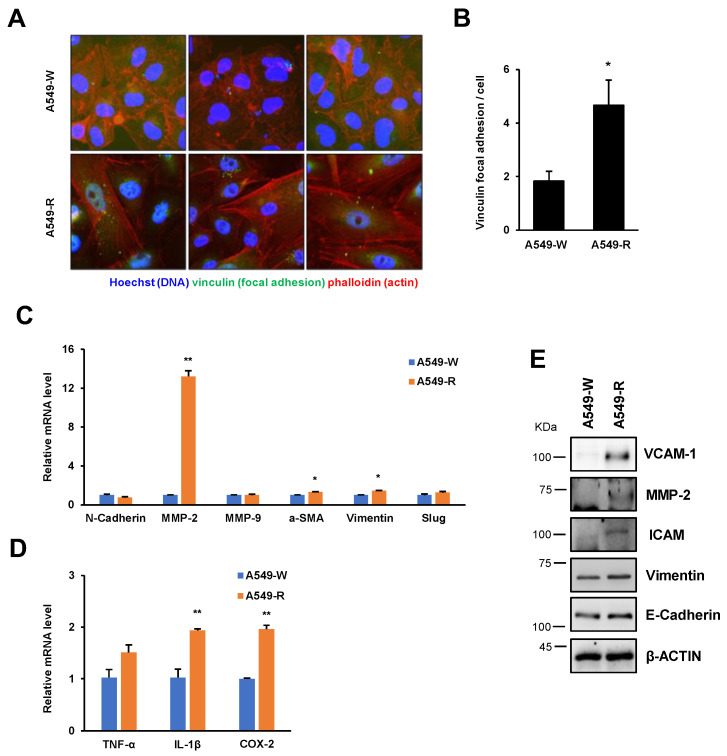
A549-R cells exhibit increased focal adhesion formation and invasive and proinflammatory factors. (**A**,**B**) Stress fiber and focal adhesion formation of A549-R cells were measured by immunofluorescence assay. Cells were immunoassayed using antibody to vinculin and 488 dye-labeled secondary antibody, further stained with 594 phalloidin and Hoechst, and then visualized by fluorescence microscopy. (**C**,**D**) RT-qPCR of proinflammatory and cell adhesion genes expressed in both A549-W and A549-R cells. (**E**) Whole-cell lysates were analyzed by Western blotting using the antibodies indicated on the right. β-ACTIN was used as a loading control. Band densitometry was analyzed for quantification, as shown in Figure S7. Quantitative data are presented as means ± SEM. Statistical comparison between groups was performed using Student’s *t*-test. The A549-W line was compared to the A549-R line in three biological replicates (*n* = 3). * *p* < 0.05, ** *p* < 0.01.

We generated volcano plots of changes in gene expression using RNA-seq data with and without metformin treatment. Notably, genes upregulated by metformin resistance were found to encode proinflammatory cytokines and cell adhesion molecules, including *BMP5*, *CXCL3*, *VCAM1*, and *POSTN* (Figure 4A). The FPKM values for representative upregulated genes are shown in Figure 4B. The FPKM values for epigenetic modifying enzyme and *ABCB* genes did not change (Figure S6). To confirm this, we performed RT-qPCR, which showed that metformin resistance markedly induced proinflammatory cytokine and cell adhesion gene expression (Figure 5A). Since metformin resistance increases the expression of proinflammatory and ECM-related genes, we next examined cell motility using a scratch wound healing assay. Compared to A549-W, A549-R showed increased wound healing (Figure 5B). We also have performed an immunofluorescence assay to test alterations in stress fiber and focal adhesion formation induced by metformin resistance. Indeed, A549-R showed increased stress fiber and focal adhesion formation, as revealed by more densely stained vinculin, which mediates interactions between integrins and the actin cytoskeleton (Figure 6A,B) [20]. Furthermore, key invasive and proinflammatory factors such as MMP2 (matrix metalloproteinase 2), VCAM (vascular cell adhesion molecule 1), and COX2 (cyclooxygenase 2) were interestingly increased in A549-R compared to A549-W (Figure 6C–E and Figure S7) [21]. These observations are consistent with previous RNA-seq and RT-qPCR data, supporting the hypothesis that metformin resistance may advance cancer cell invasion. This indicated that proinflammatory and cell motility-related genes are highly induced in metformin-resistant cancer cells.

### 3.4. A549-R Cells Show Various Cancer Cell Phenotype Conversions

**Figure 7:**
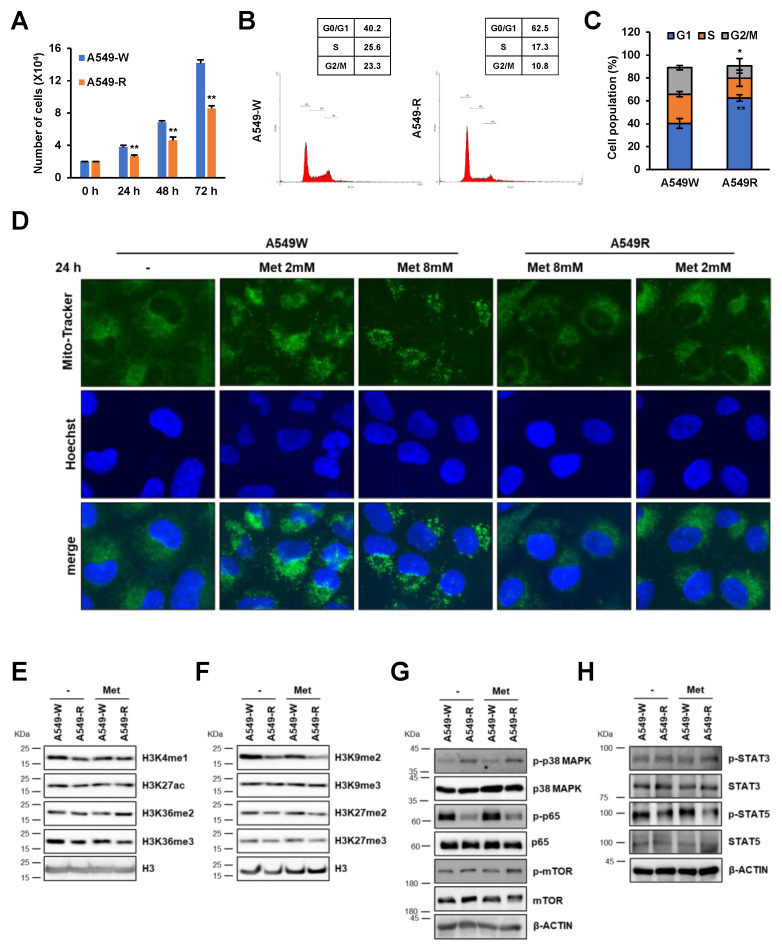
A549-R cells show various cancer cell phenotype conversions. (**A**) A549-W and A549-R were seeded in 6-well plates, and cell growth rates were analyzed by trypan blue exclusion assay. (**B**,**C**) Cells were seeded, followed by cell cycle analysis using propidium iodide. The DNA content profiles were determined by flow cytometry. (**D**) Cells were pretreated with or without 2, 8 mM metformin for 24 h, followed by treatment with Mito-Tracker Green and Hoechst 33342 dye for 1 h. Mitochondria and nuclei were observed by fluorescence microscopy. (**E**–**H**) A549-W and A549-R were treated with 2 mM metformin for 24 h, followed by histone or whole-cell lysate extraction. Histone extracts (**E**,**F**) from A549-WT or A549-R were analyzed by Western blotting using the antibodies indicated on the right. Representative activation (**E**) and repression (**F**) histone marks are shown. H3 was used as the loading control. To screen representative kinases for cell signaling, whole-cell lysates (**G**,**H**) were analyzed by Western blotting using the antibodies indicated on the right. β-ACTIN was used as a loading control. Band densitometry was analyzed for quantification, as shown in Figure S9. Statistical comparison between groups was performed using Student’s *t*-test. The A549-W line was compared to the A549-R line in three biological replicates (*n* = 3). * *p* < 0.05, ** *p* < 0.01.

Numerous studies have demonstrated that metformin exerts a positive effect on various cancers by reducing incidence and mortality [8]. To test the effect of additional drug-resistant phenotypes on A549-R, we examined growth rates, cell cycle alterations, and changes in mitochondrial integrity. Metformin-resistant A549-R cells showed growth retardation compared to A549-W (Figure 7A). Notably, G1-phase cell cycle arrest was higher in A549-R cells than in A549-W (Figure 7B,C), but dramatic changes were not observed in the expression of representative cell cycle regulators (Figure S8). Moreover, metformin treatment markedly induced mitochondrial fragmentation in A549-W cells but not in A549-R (Figure 7D). These observations indicated that metformin resistance could lead to various cancer cell phenotype conversions, including those of cell cycle and mitochondrial integrity.

An earlier study reported that metformin treatment leads to histone modifications such as acetylation and methylation via AMPK/PGC-1α in human placental explants [22]. We hypothesized that metformin resistance affects histone modification and investigated the effect of metformin resistance on histone H3 modification in A549-W and A549-R cells. First, we measured active histone marks of H3 modifications, including H3K4me1, H3K27ac, H3K36me2, and H3K36me3, and found that they were not altered by metformin resistance or treatment (Figure 7E). Notably, among the repressive histone H3 marks, including H3K9 and H3K27 methylation, the level of H3K9me2 was specifically lower in A549-R than in A549-W (Figure 7F), suggesting that metformin resistance may affect gene expression by impairing transcriptional regulation. Metformin inhibits various intracellular signaling pathways, such as p38 MAPK, NF-kB, mTOR, and STATs in cancer cells [23]; hence, we further examined the alteration of signaling pathways in A549-R. Notably, phosphorylation of p38 MAPK was increased by metformin resistance, but that of NF-kB p65 was decreased (Figure 7G,H and Figure S9). Indeed, the p38 MAPK inhibitor, but not rapamycin, attenuated up-regulation of the *VCAM* gene by metformin resistance (Figure S10). Together, these results indicated that metformin resistance might affect the key phenotypes of cancer cells in an epigenetic- or signaling pathway-dependent manner.

## 4. Discussion

We established metformin-resistant A549-R cells to determine the side effects of metformin resistance. Metformin is mainly used as a combination therapy, and sometimes cancer cells with acquired resistance show cross-resistance to other chemotherapeutic drugs, such as tyrosine kinase inhibitors [24]. The representative FDA-approved EGFR inhibitor gefitinib (the brand name Iressa) is a medication used for non-small cell lung cancers [17]. However, A549-R did not show cross-resistance to gefitinib. The other cancer cell lines, MDA-MB-231 and HeLa, showed a relatively higher IC50 of metformin treatment compared to A549 lung cancer cells, suggesting that A549 is a suitable cancer cell model for the resistance studies. Unbiased transcriptome analysis of these cells revealed that proinflammatory and invasive genes were induced by metformin resistance. Metformin resistance induced cell migration, focal adhesion formation, and G1-phase cell cycle arrest but inhibited mitochondrial fragmentation. We demonstrated that the upregulation of invasive and inflammatory genes could be a crucial cellular event for metformin resistance in cancer cells. Careful prescription of metformin could minimize resistance and side effects in patients with lung cancer.

Metformin is the most widely used drug for treating diabetes, prediabetes, polycystic ovary syndrome (PCOS), and obesity. Patients taking metformin have a lower risk of cancer, and several studies have reported that metformin may have an inhibitory effect on tumor progression [14]. More than 50 ongoing clinical trials have investigated the use of metformin in various human cancers [14]. Although metformin has well-known anti-cancer properties, such as cell cycle arrest and apoptotic cell death, its side effects and the development of resistance upon long-term treatment are unclear.

Some studies have shown that metformin treatment has no significant anti-tumor effect in several cancer types, including endometrial, bladder, thyroid, lung, and prostate cancers [25,26,27,28,29]. For example, although preclinical studies have shown that metformin can sensitize lung cancer cells to tyrosine kinase inhibitors (TKIs), adding metformin to TKI therapy resulted in worse outcomes and increased toxicity in patients with non-small cell lung cancer [17]. These observations indicate that long-term administration of metformin may be safe, but resistance to metformin in tumors may occur. Interestingly, microarray analysis by an in vitro study has shown that metformin resistance appears to trigger a transcriptome reprogramming toward a metastatic stem-like gene expression profile in MCF-7 breast cancer cells [30]. These results indicate the potential for similar metformin resistance to occur in different types of cancer. In this study, we sought to establish and characterize the A549-R cell line to determine the effects of metformin resistance. We established the cell line and examined changes in gene expression, cell migration, cell cycle, and mitochondrial fragmentation. Our results demonstrated that metformin drug resistance increases the expression of proinflammatory and invasive genes, including *BMP5*, *CXCL3*, *VCAM1*, and *POSTN*, in A549 lung cancer cells. A549 is a well-established human lung adenocarcinoma cell line that contains wild-type *EGFR* (epidermal growth factor receptor) but a homozygous *G12S KRAS* mutation [31]. Further studies are needed to test metformin resistance in other cell line model systems with different EGFR and KRAS oncogene backgrounds, such as EGFR and KRAS mutations.

Furthermore, we found that metformin resistance leads to increased cell migration and G1-phase cell cycle arrest but impairs metformin-induced mitochondrial fragmentation. Since mitochondria are one of the most crucial target organelles of metformin [32], mitochondrial fragmentation may be a sign of mitochondrial-mediated cell death by metformin. This is consistent with our A549-R cells being less responsive to metformin-induced mitochondrial fragmentation. One study reported that metformin inhibits the proliferation of MCF7 breast cancer cells via G0/G1 cell cycle arrest [33]. Metformin also could inhibit TGF-β/PI3K/AKT signaling, leading to cell cycle arrest and inhibiting colorectal cancer growth [34]. G1 cell cycle arrest may be required for cancer cell migration; for example, it is well known that TGF-β causes G1-phase cell cycle arrest but plays a vital role in cell migration undergoing epithelial-mesenchymal transition (EMT) [35]. However, we did not observe any significant changes in the expression of genes associated with glucose utilization, mitochondrial metabolism, and DNA damage in our bulk RNA-seq data, possibly due to the heterogeneity of the A549-R cell line. New perspectives are emerging on tumor heterogeneity and drug resistance mechanisms as important in lung cancer targeted therapy [36]. Thus, future work will be needed to generate metformin-resistant single-clone cells and identify gene expression changes through single-cell analysis.

Metformin is an anti-hyperglycemic agent with systemic effects on organs and tissues such as the liver, adipose tissue, muscle, and pancreas. It contributes to a decrease in hepatic gluconeogenesis, lipid synthesis, and insulin secretion by targeting glucose homeostasis and insulin signaling in these metabolic tissues [37]. In particular, the ability of metformin to inhibit mitochondrial respiratory chain complex 1 indicates its potential use as an anti-tumor agent [38]. One study has reported that mitochondrial ABCB1 (ATP binding cassette subfamily B member 1, also known as multidrug protein 1) plays a key role in the chemoresistance to metformin in human malignant mesothelioma [39]. Although metformin is known to decrease mitochondrial oxygen consumption and subsequent energy charge, it does not act by a single unifying mechanism, such as AMPK activation. Recent studies have shown that metformin can target cancer cells through epigenetic alterations, such as DNA methylation and histone modification [14]. Several in vitro studies have shown that metformin affects global DNA methylation [40,41], histone acetylation [42], and histone methylation [43,44] in cancer cells. Similarly, we found that metformin resistance selectively reduced global levels of H3K9me2 but not H3K27 acetylation and other histone methylations critical for gene expression. Histone deacetylase inhibitors have been reported to sensitize renal cell carcinoma cells through histone H3 acetylation [16]. However, we could not see significant gene expression changes on groups of genes associated with ATP binding cassette subfamily and epigenetic modifying enzymes from our bulk RNA-seq data. Thus, future work will be needed to clarify epigenetic alterations caused by long-term metformin administration or metformin resistance in anti-cancer and -diabetic treatment.

In summary, our findings suggest that the development of metformin resistance during cancer treatment with metformin may potentially lead to invasive phenotype conversion. Thus, long-term administration of metformin may result in various unexpected effects, inducing cancer cell invasion, cell cycle changes, and mitochondrial alterations. Further studies are needed to determine whether metformin resistance can emerge in patients with cancer in vivo. Furthermore, the context-dependent effect of metformin resistance in other tumor types requires further study. Notably, the expression of proinflammatory and invasive genes was markedly induced in metformin-resistant A549 cells. Further clinical and animal studies are required to investigate metformin resistance in patients with cancer.

## Data Availability

Summarized RNA-seq data are presented in Table S2, and the raw data can be provided on request.

## Supplementary Materials

The following supporting information can be downloaded at: https://www.mdpi.com/article/10.3390/genes14051014/s1, Figure S1: Establishment of metformin-resistant A549 human lung cancer cells (A549-R cells); Figure S2: A549-R cells did not show cross-resistance to gefitinib; Figure S3: Cell viability of representative cancer cell lines to metformin treatment; Figure S4: Inter-sample correlation heat map of A549-R cells upon metformin treatment; Figure S5: Gene ontology analysis of A549-R cells upon metformin treatment; Figure S6: Representative FPKM values of epigenetic modifying enzyme and ABCB genes in A549-R cells upon metformin treatment; Figure S7: A549-R cells exhibit increased invasive and focal adhesion factors; Figure S8: A549-R cells show little effect on expression of cell cycle regulators; Figure S9: Alterations of epigenetic histone marks and signaling pathways in A549-R cells; Figure S10: p38 MAPK inhibitor, but not rapamycin, attenuated up-regulation of VCAM gene by metformin-resistance; Table S1: Antibodies; Table S2: Raw RNA-seq data of A549-R following metformin treatment; Table S3; The SYBR green primers for RT-qPCR.
