# Supplementary material for: Metformin Resistance Is Associated with Expression of Inflammatory and Invasive Genes in A549 Lung Cancer Cells
Source: Genes (Basel). 2023 Apr 29;14(5):1014. doi: 10.3390/genes14051014 (PMC10218153; doi:10.3390/genes14051014)
Supplement: Supplementary file 1 [file genes-14-01014-s001.zip › A549-met_Supplementary Figure 230424 final.pptx]

## Slide 1
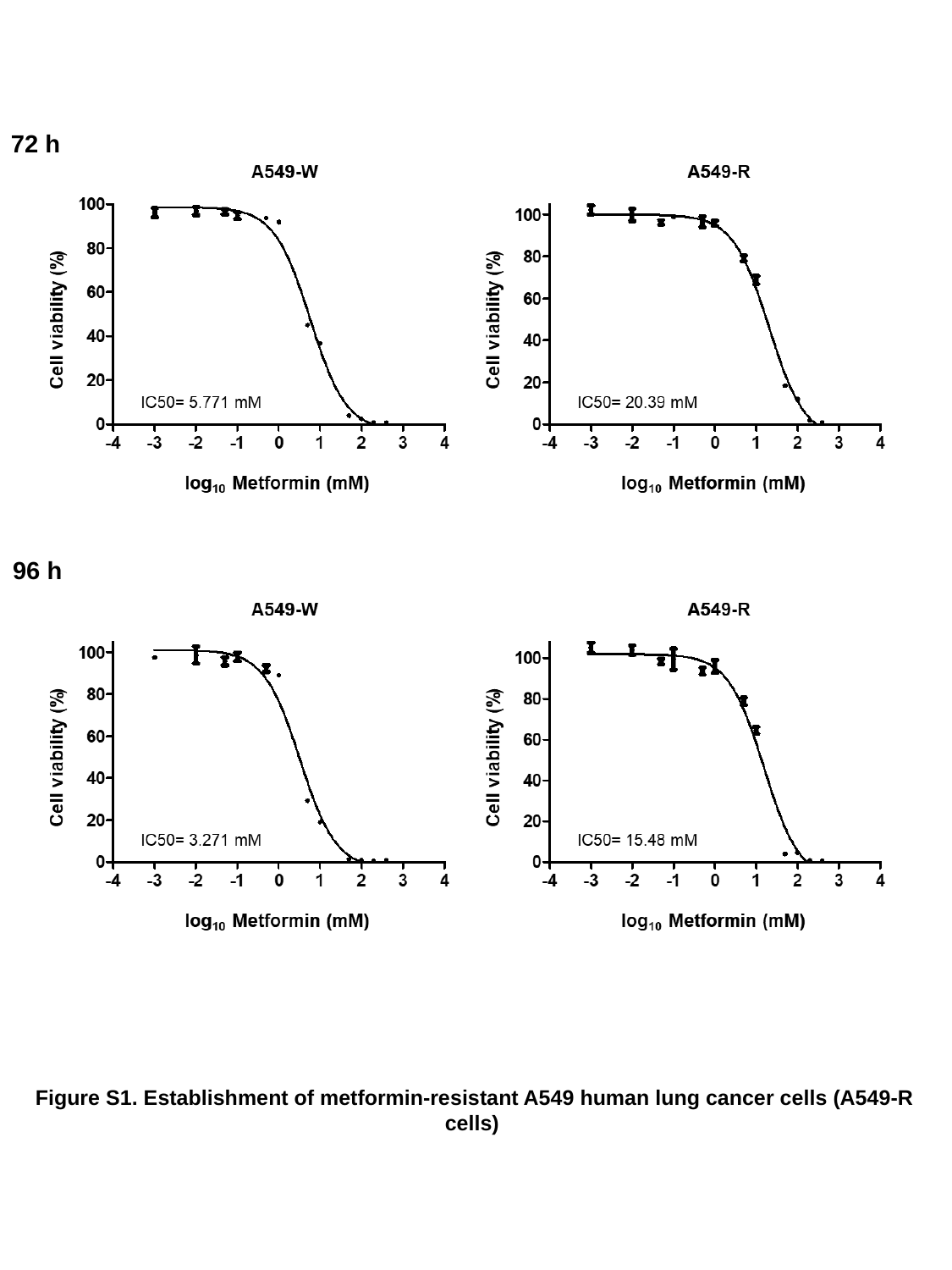

72 h
96 h
Figure S1. Establishment of metformin-resistant A549 human lung cancer cells (A549-R cells)

## Slide 2
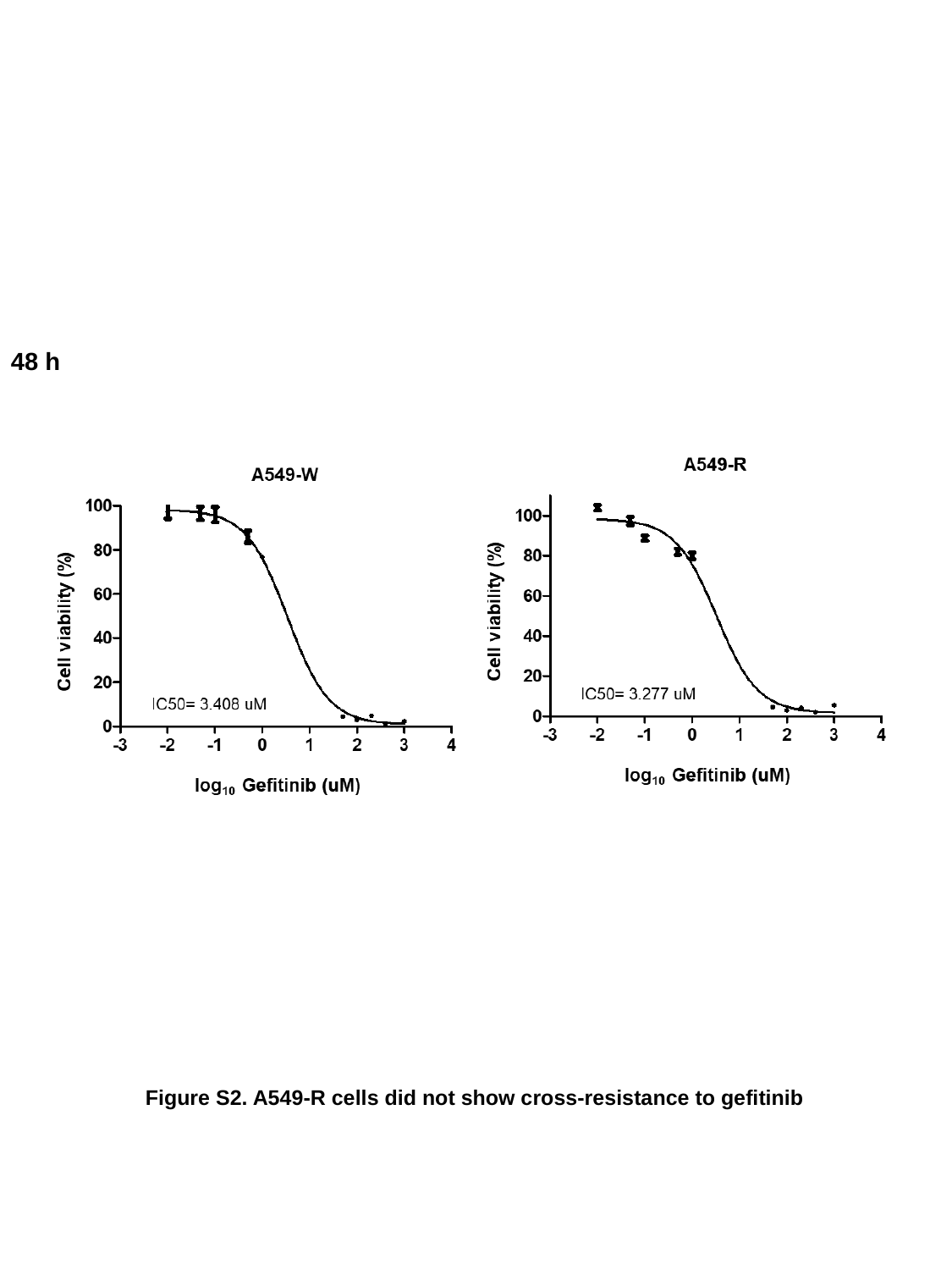

48 h
Figure S2. A549-R cells did not show cross-resistance to gefitinib

## Slide 3
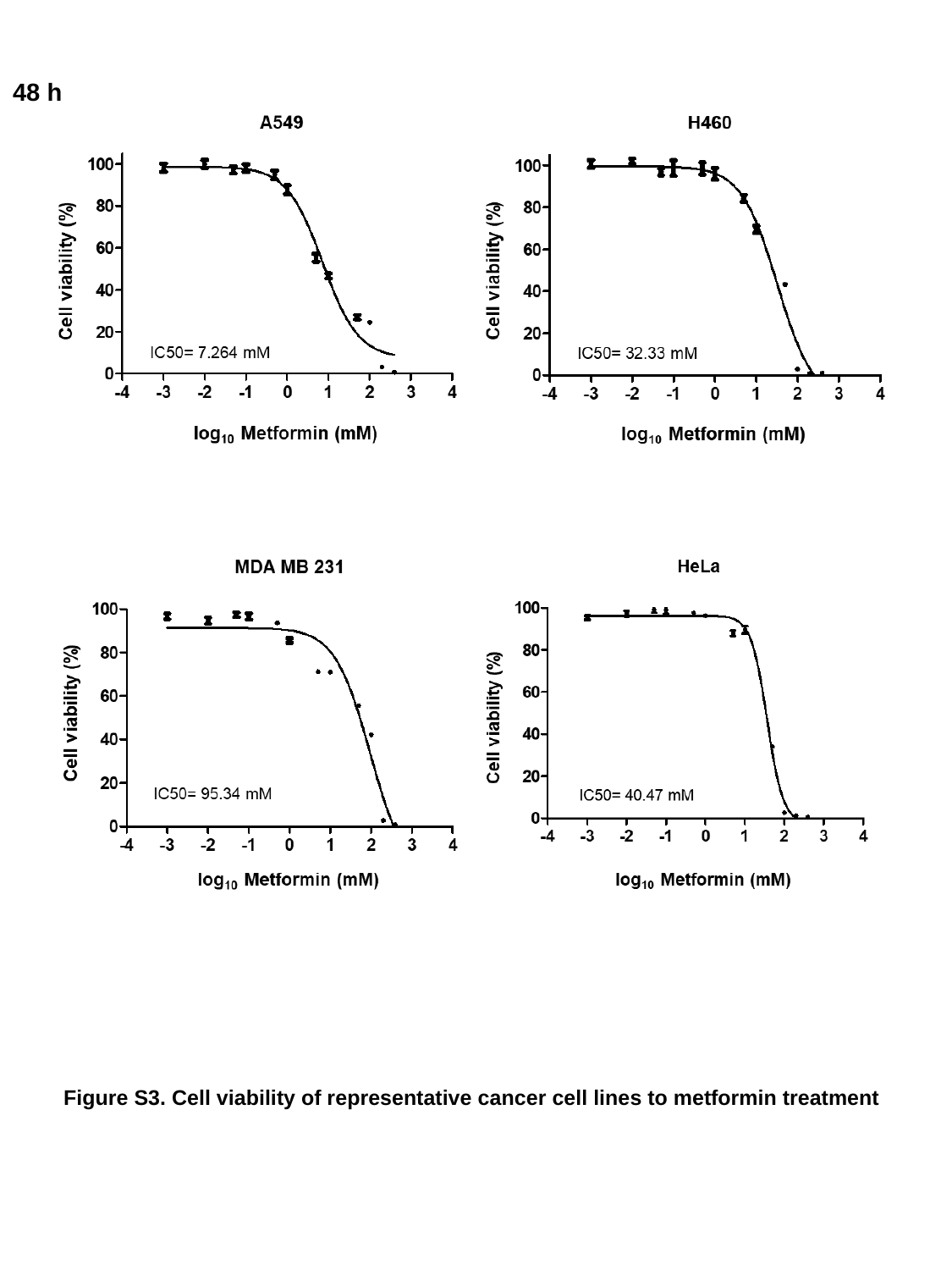

48 h
Figure S3. Cell viability of representative cancer cell lines to metformin treatment

## Slide 4
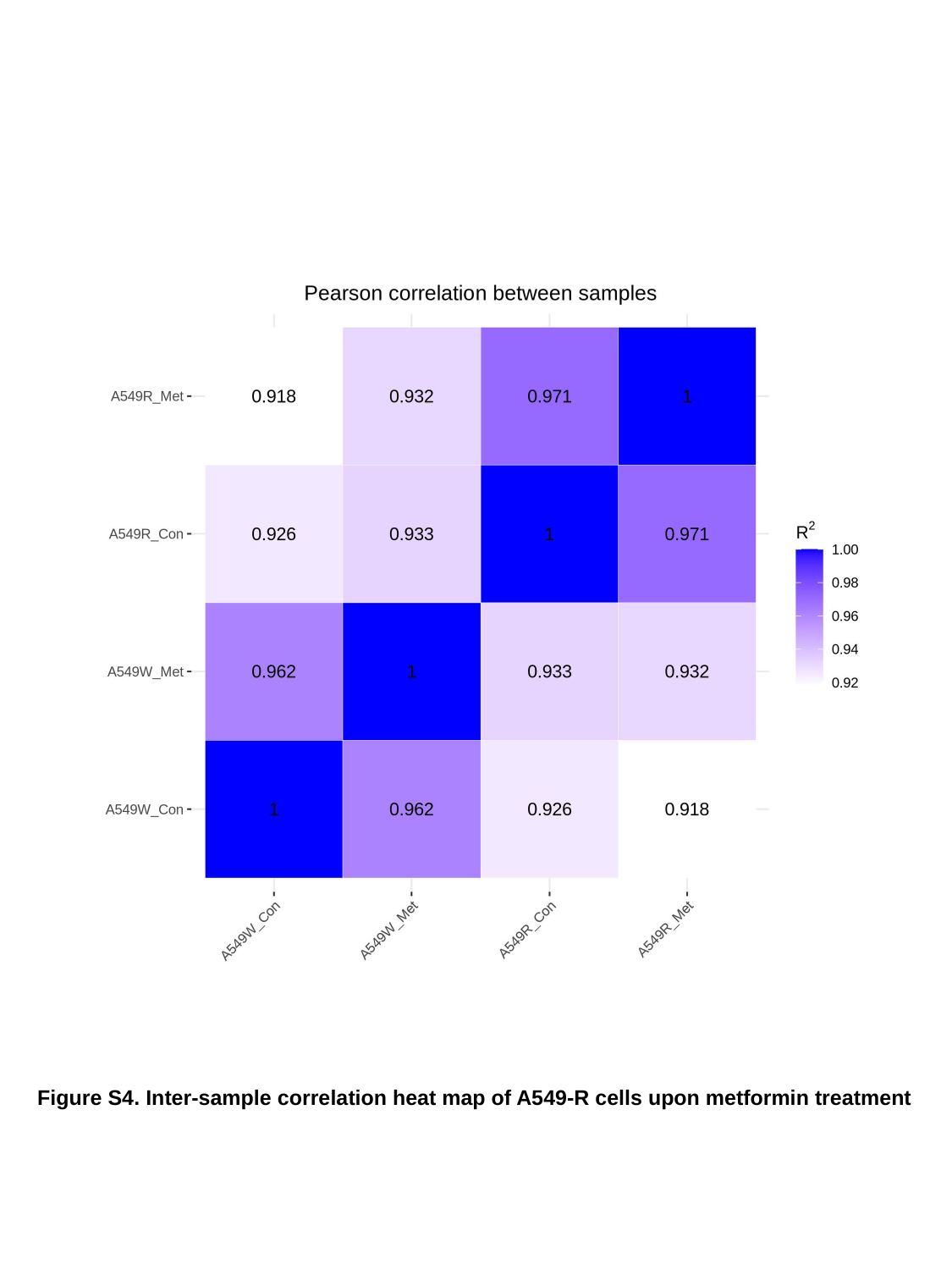

Figure S4. Inter-sample correlation heat map of A549-R cells upon metformin treatment

## Slide 5
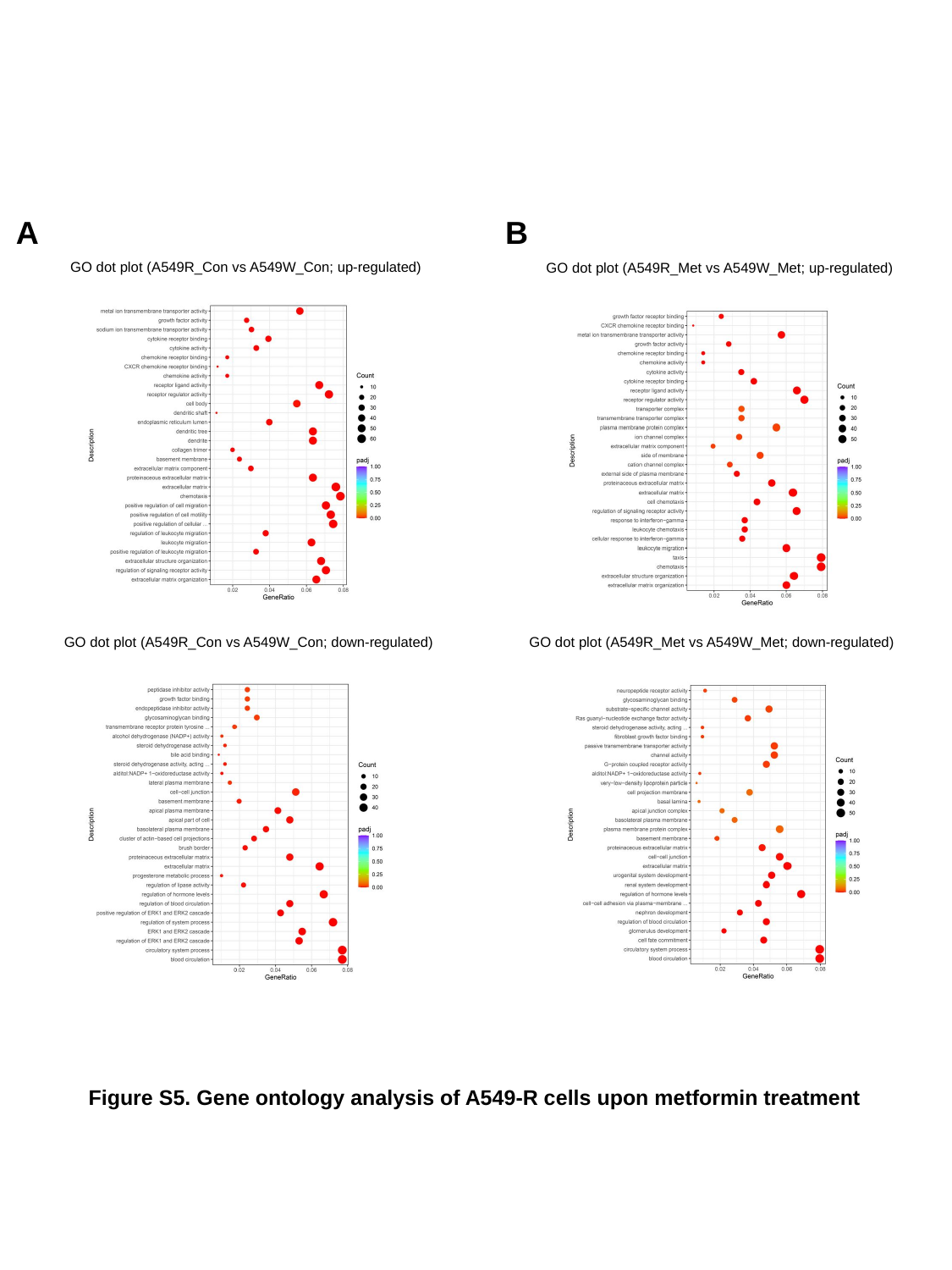

A
B
GO dot plot (A549R_Con vs A549W_Con; up-regulated)
GO dot plot (A549R_Met vs A549W_Met; up-regulated)
GO dot plot (A549R_Con vs A549W_Con; down-regulated)
GO dot plot (A549R_Met vs A549W_Met; down-regulated)
Figure S5. Gene ontology analysis of A549-R cells upon metformin treatment

## Slide 6
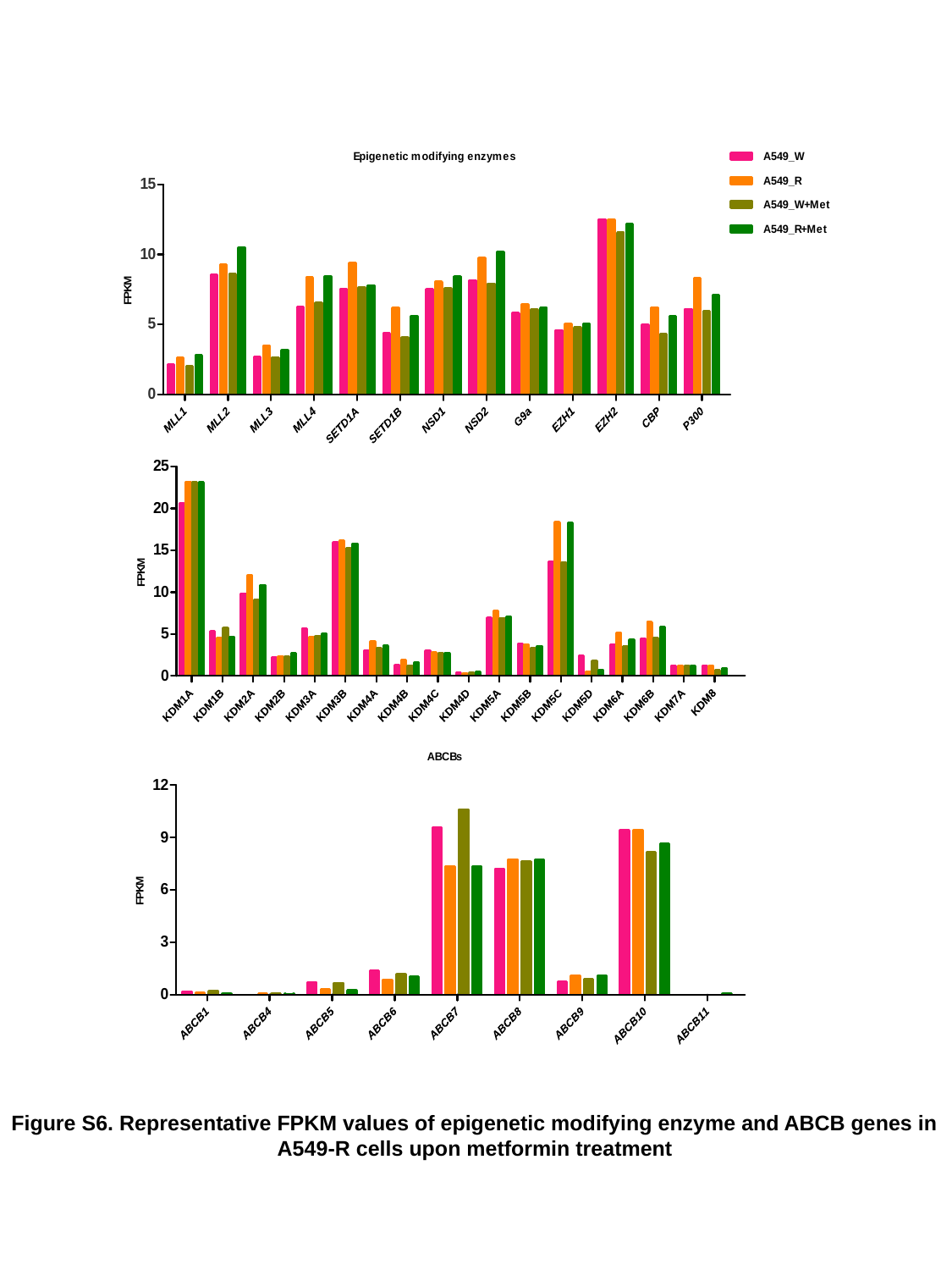

Figure S6. Representative FPKM values of epigenetic modifying enzyme and ABCB genes in A549-R cells upon metformin treatment

## Slide 7
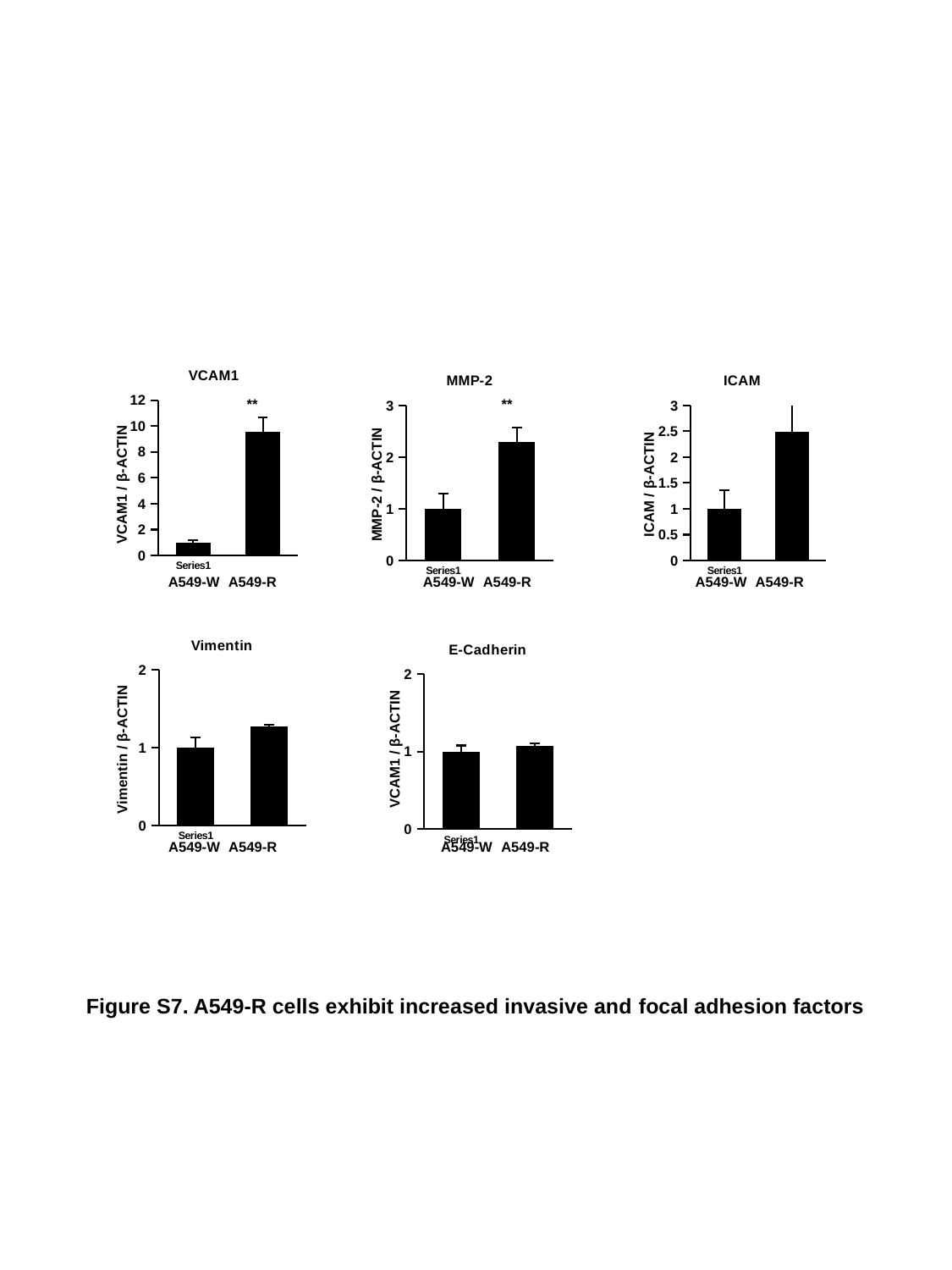

### Chart: VCAM1
| Category | |
|---|---|
| | 1.0 |
| | 9.546576749558103 |
### Chart: MMP-2
| Category | |
|---|---|
| | 1.0 |
| | 2.2890734266986463 |
### Chart: ICAM
| Category | |
|---|---|
| | 1.0 |
| | 2.481313048627772 |**
**
VCAM1 / β-ACTIN
MMP-2 / β-ACTIN
ICAM / β-ACTIN
A549-W
A549-R
A549-W
A549-R
A549-W
A549-R
### Chart: Vimentin
| Category | |
|---|---|
| | 1.0 |
| | 1.276837529378627 |
### Chart: E-Cadherin
| Category | |
|---|---|
| | 1.0 |
| | 1.0705442647583938 |Vimentin / β-ACTIN
VCAM1 / β-ACTIN
A549-W
A549-R
A549-W
A549-R
Figure S7. A549-R cells exhibit increased invasive and focal adhesion factors

## Slide 8
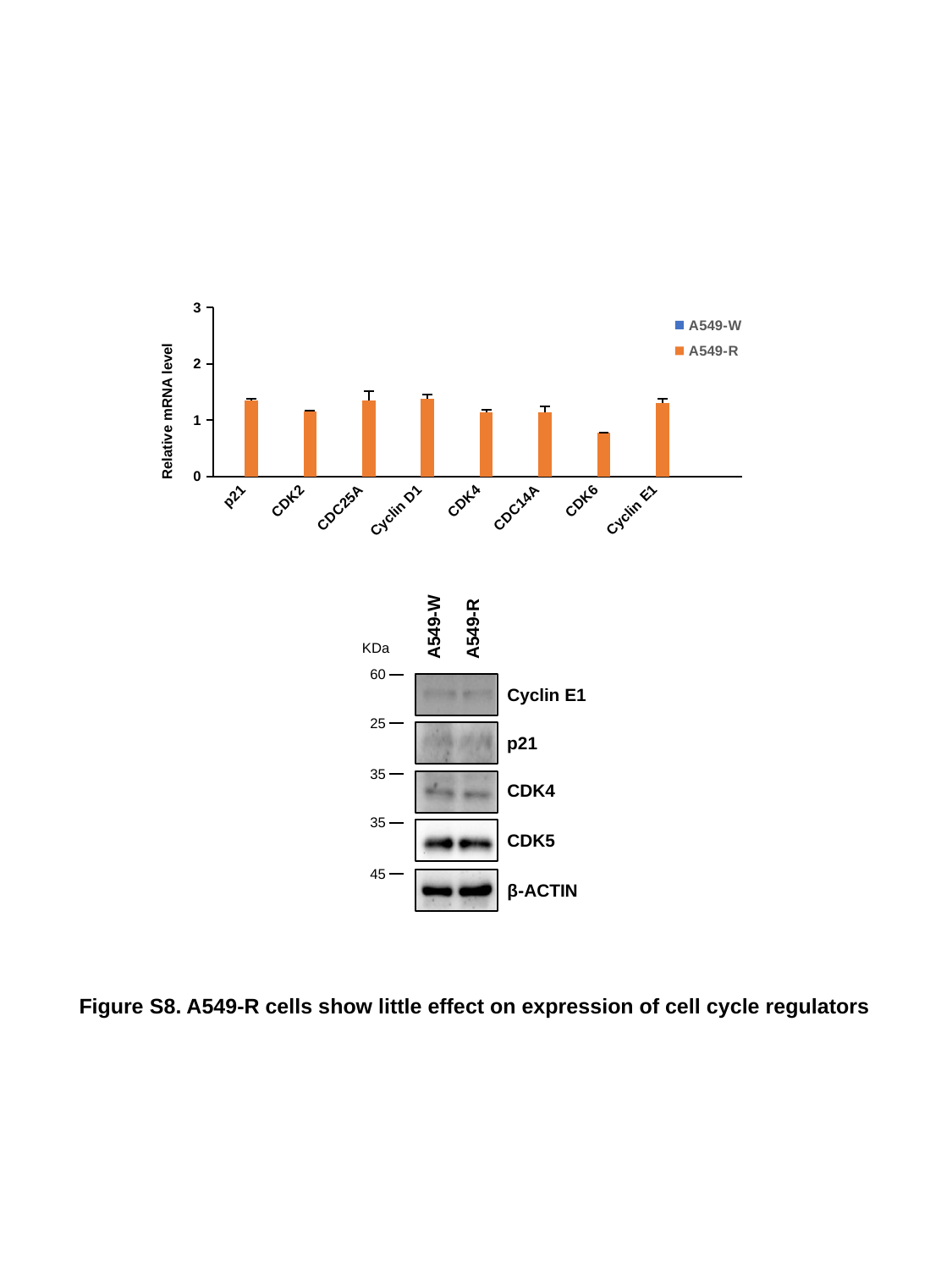

### Chart
| Category | A549-W | A549-R |
|---|---|---|
| p21 | 1.000889704691729 | 1.3447025550802867 |
| CDK2 | 1.002202965435232 | 1.1560230461903005 |
| CDC25A | 1.002512969844468 | 1.350764274168668 |
| Cyclin D1 | 1.0033943996437833 | 1.370896646700384 |
| CDK4 | 1.0023923916114619 | 1.1424171367658822 |
| CDC14A | 1.012599182879745 | 1.1408410314289186 |
| CDK6 | 1.010604637057189 | 0.7676093209533571 |
| Cyclin E1 | 1.0002071306268276 | 1.302703223766791 |Relative mRNA level
A549-W
A549-R
KDa
60
Cyclin E1
25
p21
35
CDK4
35
CDK5
45
β-ACTIN
Figure S8. A549-R cells show little effect on expression of cell cycle regulators

## Slide 9
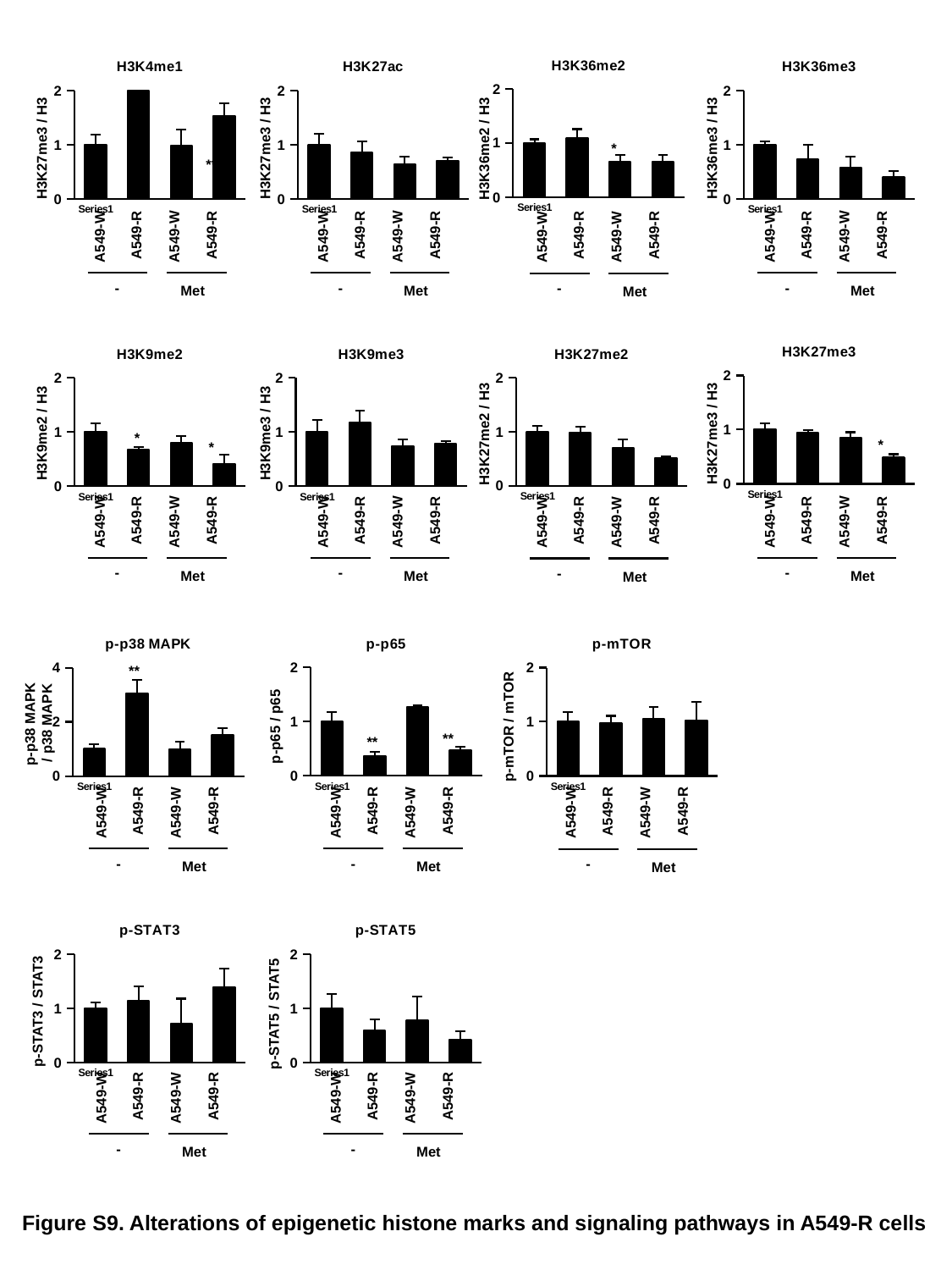

### Chart: H3K36me2
| Category | |
|---|---|
| | 1.0 |
| | 1.0946715968131782 |
| | 0.6530689825765029 |
| | 0.6585936315376316 |
### Chart: H3K4me1
| Category | |
|---|---|
| | 1.0 |
| | 3.047818435954173 |
| | 0.9733225952952065 |
| | 1.5259944138540646 |
### Chart: H3K27ac
| Category | |
|---|---|
| | 1.0 |
| | 0.854276693443811 |
| | 0.6368440296869161 |
| | 0.696015858530898 |
### Chart: H3K36me3
| Category | |
|---|---|
| | 1.0 |
| | 0.7250198091641357 |
| | 0.5700539660012467 |
| | 0.3941996153996023 |H3K27me3 / H3
H3K27me3 / H3
H3K36me3 / H3
*
H3K36me2 / H3
**
A549-R
A549-R
A549-R
A549-R
A549-R
A549-R
A549-R
A549-R
A549-W
A549-W
A549-W
A549-W
A549-W
A549-W
A549-W
A549-W
-
-
-
-
Met
Met
Met
Met
### Chart: H3K27me3
| Category | |
|---|---|
| | 0.9999999999999999 |
| | 0.9414753217631886 |
| | 0.8449619966495806 |
| | 0.4835348896528893 |
### Chart: H3K27me2
| Category | |
|---|---|
| | 1.0 |
| | 0.9817457091183904 |
| | 0.7061816480472848 |
| | 0.5072838691378585 |
### Chart: H3K9me2
| Category | |
|---|---|
| | 1.0000000000000002 |
| | 0.6748760276172758 |
| | 0.8053557243511357 |
| | 0.4136272124825369 |
### Chart: H3K9me3
| Category | |
|---|---|
| | 1.0 |
| | 1.1781726089491837 |
| | 0.738984830766643 |
| | 0.7837623403266547 |H3K9me2 / H3
H3K9me3 / H3
H3K27me3 / H3
H3K27me2 / H3
*
*
*
A549-R
A549-R
A549-R
A549-R
A549-R
A549-R
A549-R
A549-R
A549-W
A549-W
A549-W
A549-W
A549-W
A549-W
A549-W
A549-W
-
-
-
-
Met
Met
Met
Met
### Chart: p-p65
| Category | |
|---|---|
| | 1.0 |
| | 0.369628420779529 |
| | 1.2747667595223147 |
| | 0.47389927560324674 |
### Chart: p-mTOR
| Category | |
|---|---|
| | 1.0 |
| | 0.9745981852823116 |
| | 1.0581068148053923 |
| | 1.0210174905787117 |
### Chart: p-p38 MAPK
| Category | |
|---|---|
| | 1.0 |
| | 3.047818435954173 |
| | 0.9733225952952065 |
| | 1.5259944138540646 |**
p-p38 MAPK
 / p38 MAPK
p-p65 / p65
p-mTOR / mTOR
**
**
A549-R
A549-R
A549-R
A549-R
A549-R
A549-R
A549-W
A549-W
A549-W
A549-W
A549-W
A549-W
-
-
-
Met
Met
Met
### Chart: p-STAT3
| Category | |
|---|---|
| | 1.0 |
| | 1.1307931304415535 |
| | 0.7133119975998441 |
| | 1.3874023010630774 |
### Chart: p-STAT5
| Category | |
|---|---|
| | 0.9999999999999999 |
| | 0.5858029998441385 |
| | 0.7785610566500639 |
| | 0.41488493925676134 |p-STAT3 / STAT3
p-STAT5 / STAT5
A549-R
A549-R
A549-R
A549-R
A549-W
A549-W
A549-W
A549-W
-
-
Met
Met
Figure S9. Alterations of epigenetic histone marks and signaling pathways in A549-R cells

## Slide 10
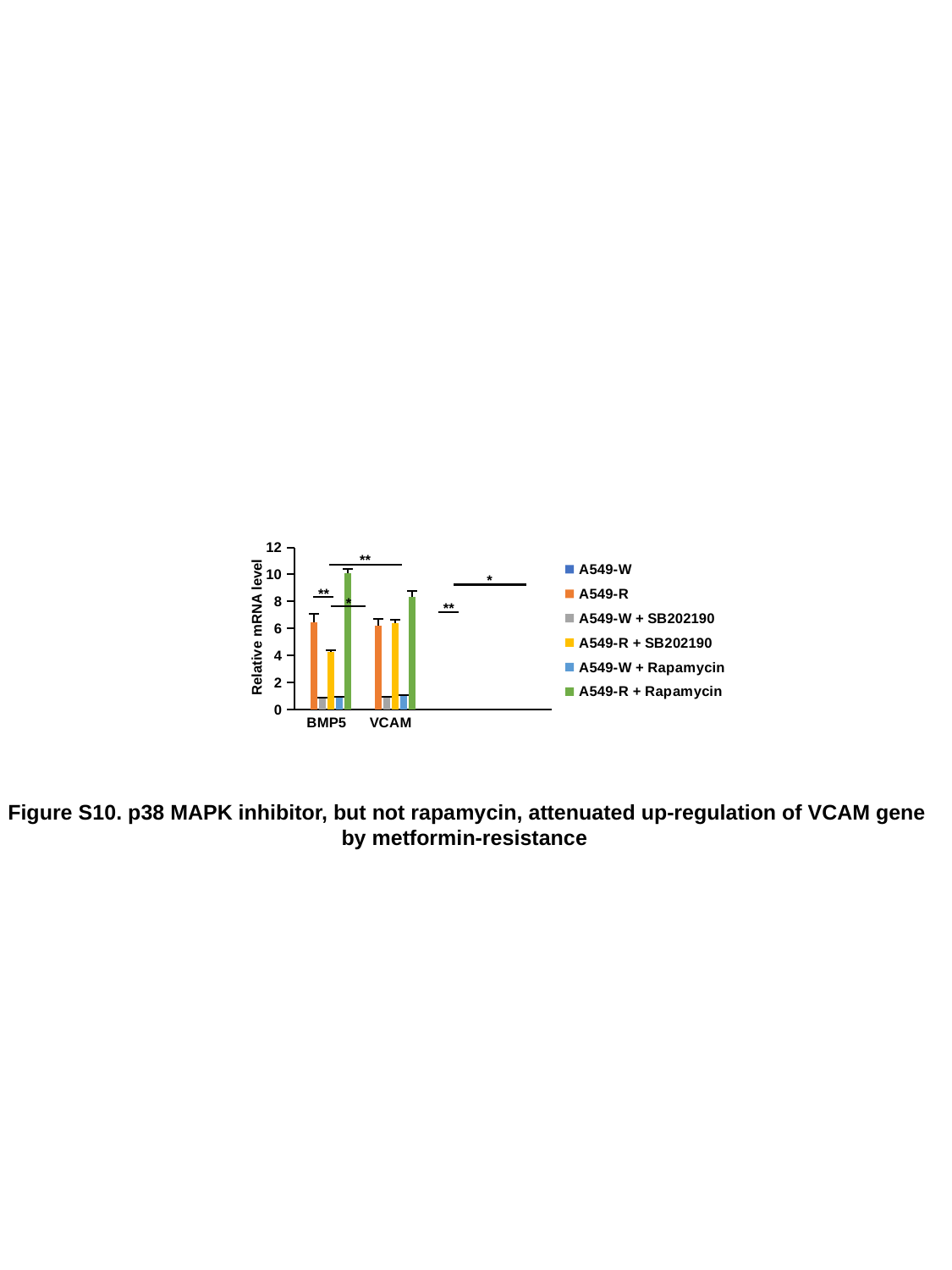

### Chart
| Category | A549-W | A549-R | A549-W + SB202190 | A549-R + SB202190 | A549-W + Rapamycin | A549-R + Rapamycin |
|---|---|---|---|---|---|---|
| BMP5 | 1.002973737261473 | 6.460019740712037 | 0.8704854881520213 | 4.278484020343151 | 0.9242016223304238 | 10.11750974294984 |
| VCAM | 1.0000849575103439 | 6.212817145283172 | 0.9126542657521806 | 6.417198791468977 | 0.9781197729795258 | 8.335790654395439 |**
*
**
*
**
Relative mRNA level
Figure S10. p38 MAPK inhibitor, but not rapamycin, attenuated up-regulation of VCAM gene by metformin-resistance
