# Supplementary material for: Metformin Resistance Is Associated with Expression of Inflammatory and Invasive Genes in A549 Lung Cancer Cells
Source: Genes (Basel). 2023 Apr 29;14(5):1014. doi: 10.3390/genes14051014 (PMC10218153; doi:10.3390/genes14051014)
Supplement: Supplementary file 1 [file genes-14-01014-s001.zip › Supplementary Table S1. Antibodies.pdf]

Table S1. Antibodies

Antibodies	SOURCE	IDENTIFIER
Anti-H3	Cell Signaling Technology	4499
Anti-H3K4me1	Cell signaling Technology	5326
Anti-H3K9me2	Cell signaling Technology	4658
Anti-H3K9me3	Cell signaling Technology	13969
Anti-H3K27me2	Cell signaling Technology	9728
Anti-H3K27me3	Cell signaling Technology	9733
Anti-H3K27ac	Cell signaling Technology	8179
Anti-H3K36me2	Cell signaling Technology	2901
Anti-H3K36me3	Cell signaling Technology	4909
Anti-p38 MAPK	Cell signaling Technology	9212
Anti-phospho-mTOR	Cell signaling Technology	5536
Anti-mTOR	Cell signaling Technology	2983
Anti-β-actin	BioLegend	622102
Anti-phospho-STAT3	BioLegend	651001
Anti-STAT3	BioLegend	678002
Anti-STAT5	BioLegend	660202
Anti-phospho-NFkB p65	Abcam	ab76302
Anti-phospho-STAT5	Santa Cruz Biotechnology	sc-101806
Anti-NFkB p65	Santa Cruz Biotechnology	sc-372
Anti-phospho-p38 MAPK	BD Biosciences	612280
Anti-MMP-2	Abcam	ab6003
Anti-vimentin	Santa Cruz Biotechnology	sc-6260
Anti-p21	Santa Cruz Biotechnology	sc-397
Anti-VCAM-1	Santa Cruz Biotechnology	sc-8304
Anti-E-cadherin	BD Biosciences	610182
Anti-cyclin E1	Santa Cruz Biotechnology	sc-25303
Anti-Rabbit HRP	GeneTex	GTX213110-01
Anti-Mouse HRP	GeneTex	GTX213110-11
Anti-Vinculin	Santa Cruz Biotechnology	sc-5573
Anti-Rabbit FSD™ 488	BioActs	RSA1245
Anti-Cdk4	Santa Cruz Biotechnology	sc-23896
Anti-Cdk5	Santa Cruz Biotechnology	sc-249
Anti-ICAM	Santa Cruz Biotechnology	sc-8439
