# Supplementary material for: Metformin Resistance Is Associated with Expression of Inflammatory and Invasive Genes in A549 Lung Cancer Cells
Source: Genes (Basel). 2023 Apr 29;14(5):1014. doi: 10.3390/genes14051014 (PMC10218153; doi:10.3390/genes14051014)
Supplement: Supplementary file 1 [file genes-14-01014-s001.zip › Supplementary Table S3. The SYBR green primers for qRT-PCR.pdf]

Table S3. The SYBR green primers for qRT-PCR

	Gene	Forward sequence	Reverse sequence
KEEG Cytokine-cytokine interaction	*TNFRSF19*	GGTGCATTCTGCAGCCAGTCTT	CAGGCATCTGAAAACTCGCCAC
	IL6	AGACAGCCACTCACCTCTTCAG	TTCTGCCAGTGCCTCTTTGCTG
	BMP5	CTCTCATCAGGACTCCTCCAGA	GGAAGCTCACATAGAGTTCGTGC
	CXCL1	AGCTTGCCTCAATCCTGCATCC	TCCTTCAGGAACAGCCACCAGT
	CXCL3	TTCACCTCAAGAACATCCAAAGTG	TTCTTCCCATTCTTGAGTGTGGC
	CXCL8	GAGAGTGATTGAGAGTGGACCAC	CACAACCCTCTGCACCCAGTTT
	CCL2	AGAATCACCAGCAGCAAGTGTCC	TCCTGAACCCACTTCTGCTTGG
	CXCL2	GGCAGAAAGCTTGTCTCAACCC	CTCCTTCAGGAACAGCCACCAA
	CXCL5	CAGACCACGCAAGGAGTTCATC	TTCCTTCCCGTTCTTCAGGGAG
	BMP7	GAGTGTGCCTTCCCTCTGAACT	AGGACGGAGATGGCATTGAGCT
	CD70	TTCGCACAGGCTCAGCAGCAG	TTGTCCAGCTCTGGTCCATGCA
	CCL26	GGGAGTGACATATCCAAGACCTG	CAGACTTTCTTGCCTCTTTTGGTA
GO Cell adhesion	*COL15A1*	GGTGACACTGGTTTACCTGGCT	GCCTTTCCAGAGGAATGTCCTC
	LAMA4	GAGATGACTCTCTGCTGGACCT	AGTTCCAGGCAGCCAACAAAGC
	TNC	ATGTCCTCCTGACAGCCGAGAA	AGTCACGGTGAGGTTTTCCAGC
	EPHA4	ACCAAGCAGTGCGAGAGTTTGC	CTCTCTTGCCAGGCACTTTGAG
	ADAM12	ATGGCATCTGCCAGACTCACGA	GGAACTCTTCGAGACTTTGCCAC
	ITGA4	GCATACAGGTGTCCAGCAGAGA	AGGACCAAGGTGGTAAGCAGCT
	VCAM1	GATTCTGTGCCCACAGTAAGGC	TGGTCACAGAGCCACCTTCTTG
	PCDH10	TCTCCAACGGAAGCATTTTGTCC	CTATGTCGGCTTCCTGGAATGC
	NID2	GAGCTGTACCACTACTCCGACT	TGTTCTGGTGGATGCGGTAGGA
	POSTN	CAGCAAACCACCTTCACGGATC	TTAAGGAGGCGCTGAACCATGC
	ADGRE1	TGTGACGTTGGACTTGGTAGCC	GGAGACAAAAGCCACACCAGTG
	ICAM1	AGCGGCTGACGTGTGCAGTAAT	TCTGAGACCTCTGGCTTCGTCA
	MCAM	ATCGCTGCTGAGTGAACCACAG	CTACTCTCTGCCTCACAGGTCA
	KITLG	CTGGAGACTCCAGCCTACACTG	CTGCCCTTGTAAGACTTGGCTG
	LGALS3BP	GGCAGCAATGTCACCATGAGTG	GTGGAAGCACTTGACTGACGAC
Migration / Invasion	*N-Cadherin*	CCTCCAGAGTTTACTGCCATGAC	GTAGGATCTCCGCCACTGATTC
	MMP2	AGCGAGTGGATGCCGCCTTTAA	CATTCCAGGCATCTGCGATGAG
	MMP9	GCCACTACTGTGCCTTTGAGTC	CCCTCAGAGAATCGCCAGTACT
	a-SMA	CTATGCCTCTGGACGCACAACT	CAGATCCAGACGCATGATGGCA
	Vimentin	AGGCAAAGCAGGAGTCCACTGA	ATCTGGCGTTCCAGGGACTCAT
	Slug	ATCTGCGGCAAGGCGTTTTCCA	GAGCCCTCAGATTTGACCTGTC
Cell cycle	*p21*	AGGTGGACCTGGAGACTCTCAG	TCCTCTTGGAGAAGATCAGCCG
	CDK2	ATGGATGCCTCTGCTCTCACTG	CCCGATGAGAATGGCAGAAAGC

	CDC25A	TCTGGACAGCTCCTCTCGTCAT	ACTTCCAGGTGGAGACTCCTCT
	Cyclin D1	TCTACACCGACAACTCCATCCG	TCTGGCATTTTGGAGAGGAAGTG
	CDK4	CCATCAGCACAGTTCGTGAGGT	TCAGTTCGGGATGTGGCACAGA
	CDC14A	TAGATGGCAGCACACCCAGTGA	GTCCCTGTTCTTCCAAGACCAG
	CDK6	GGATAAAGTTCCAGAGCCTGGAG	GCGATGCACTACTCGGTGTGAA
	Cyclin E1	TGTGTCCTGGATGTTGACTGCC	CTCTATGTCGCACCACTGATACC
Inflammation	*TNFa*	CTCTTCTGCCTGCTGCACTTTG	ATGGGCTACAGGCTTGTCACTC
	IL-1b	CCACAGACCTTCCAGGAGAATG	GTGCAGTTCAGTGATCGTACAGG
	COX2	CGGTGAAACTCTGGCTAGACAG	GCAAACCGTAGATGCTCAGGGA
